# CCN1 promotes IL-1β production in keratinocytes by activating p38 MAPK signaling in psoriasis

**DOI:** 10.1038/srep43310

**Published:** 2017-03-07

**Authors:** Yue Sun, Jie Zhang, Tianhang Zhai, Huidan Li, Haichuan Li, Rongfen Huo, Baihua Shen, Beiqing Wang, Xiangdong Chen, Ningli Li, Jialin Teng

**Affiliations:** 1 Department of Rheumatology and Immunology, Ruijin Hospital, Shanghai Jiao Tong University School of Medicine, Shanghai, China; 2 Shanghai Institute of Immunology and Department of Immunology and Microbiology, Shanghai Jiao Tong University School of Medicine, Shanghai, China; 3 Department of Dermatology, Ninth People’s Hospital, Shanghai Jiao Tong University School of Medicine, Shanghai, China

## Abstract

CCN1, an extracellular protein also known as cysteine-rich protein 61 (Cyr61), is a novel pro-inflammatory factor involved in the pathogenesis of rheumatoid arthritis. As an inflammatory disease, psoriasis is characterized by keratinocyte activation-induced epidermal hyperplasia and cytokine-mediated inflammation. We demonstrated in our previous study that CCN1 promoted keratinocyte activation in psoriasis. However, the role of CCN1 in regulating inflammation in psoriasis is still unknown. Here, we showed that CCN1 increased inflammatory cytokine IL-1β production in keratinocytes. Furthermore, endogenous ATP and caspase-1 were required for mature IL-1β production stimulated by CCN1 in keratinocytes. After binding to the receptor of integrin α6β1, CCN1 activated the downstream p38 MAPK signaling pathway, thus inducing the expression of IL-1β. In addition, we inhibited CCN1 function in mouse models of psoriasis, and decreased IL-1β production was observed *in vivo*. Overall, we showed that CCN1 increased IL-1β production via p38 MAPK signaling, indicating a role for CCN1 protein in regulating inflammation in psoriasis.

Psoriasis is a common chronic inflammatory disease that affects the skin and joints^1^,^2^. Although the pathogenesis of psoriasis is still not fully understood, studies have demonstrated a critical role of immune cells and cytokine-mediated (such as IL-1β, TNF-α, IL-17 and IL-23) inflammation in the development of psoriasis^1^,^3^. Another key characteristic of psoriasis is the dysregulation of keratinocyte activation, which leads to the epidermal hyperplasia^4^,^5^. Thus, the amplification cycle of keratinocyte activation and inflammation is a hallmark of psoriasis pathology.

As a highly inflammatory cytokine, interleukin-1β (IL-1β) is predominantly produced by monocytes, dendritic cells (DCs) and macrophages, but keratinocytes can also produce low amounts of IL-1β^6^,^7^. IL-1β has diverse functions, including T-cell activation, antigen recognition, Th17 cell development and IL-17 and IL-22 production by nature killer T cells (NKT) and NK cells^8^,^9^,^10^. The potent inflammatory role of IL-1β has been linked to diseases such as psoriasis and rheumatoid arthritis (RA)^11^,^12^. In psoriasis, IL-1 is believed to be the initiator of inflammation and keratinocyte activation^13^. IL-1 mediates the expression of keratin 6 and keratin 16, which are crucial for initiating and maintaining keratinocyte activation in psoriasis^14^,^15^.

It is well-established that the IL-1β precursor (pro-IL-1β, 31 kDa) is not biologically active and require proteolytic processing for activation^7^. Upon stimulation, inactive pro-IL-1β is cleaved into mature IL-1β (17 kDa) by caspase-1, which is activated by the inflammasome^16^. Caspase-1 activation is induced from exogenous or endogenous agents^17^, and adenosine triphosphate (ATP) is a well-known endogenous agonist for caspase-1-mediated IL-1β post-translational processing^7^,^16^,^17^.

CCN1, also known as cysteine-rich protein 61 (Cyr61), belongs to the CCN family^18^. CCN1 is produced by stromal cells, such as fibroblasts, epithelial cells, endothelial cells, cancer cells, and some types of immune cells^19^,^20^,^21^. CCN1 regulates cell proliferation, migration, adhesion, angiogenesis, tumorigenesis and inflammation^22^,^23^,^24^,^25^. CCN1 exerts its multiple functions predominantly by binding to various integrin receptors, leading to the activation of downstream signaling pathways, including the phosphatidylinositol 3-kinase (PI3K)/Akt and mitogen-activated protein kinase (MAPK) pathways^22^,^24^,^26^,^27^. CCN1 has been shown to enhance the synovium hyperplasia and inflammation involved in RA^24^,^26^,^28^,^29^. Interestingly, we reported that CCN1 was up-regulated in psoriatic skin lesions and promoted keratinocyte (KC) proliferation and activation via the α6β1/PI3K/Akt/NF-κB pathway in psoriasis, indicating that CCN1 was involved in the development and pathogenesis of psoriasis^27^,^30^. However, the role of CCN1 in regulating inflammation in psoriasis is still unclear. Given that IL-1β functions as an initiator and effector for inflammation and skin lesions in psoriasis, we investigated whether CCN1 contributes to IL-1β production in keratinocytes.

In this study, we aimed to elucidate the physiological function of CCN1 with regard to IL-1β production in keratinocytes. First, an *in vitro* study showed that CCN1 promoted inflammatory cytokine IL-1β production in keratinocytes. Furthermore, we found that endogenous ATP and caspase-1 were crucial for the mature IL-1β production in CCN1-stimulated keratinocytes. Then, we detected the signaling pathways involved in CCN1-induced IL-1β expression and showed that CCN1 stimulation activated the α6β1/p38 MAPK pathway. To confirm these results, we blocked CCN1 function with a monoclonal antibody in imiquimod (IMQ)-induced psoriasis-like mice or with a lentiviral vector expressing short hairpin RNA (shRNA) in IL-23-induced psoriasis-like mice, and we also found impaired IL-1β expression *in vivo*. Thus, we concluded that CCN1 increased IL-1β production via p38 MAPK signaling in keratinocytes, suggesting that CCN1 might be a potential target for ameliorating inflammation in psoriasis.

## Results

### Blocking CCN1 expression in KCs inhibited IL-1β production

In our previous study, we found that CCN1 was highly expressed in the lesional skins of psoriasis patients and was involved in the pathogenesis of psoriasis by promoting keratinocyte activation^27^. As CCN1 is considered as a pro-inflammatory factor in RA pathology, we further explored whether CCN1 contributed to the inflammation of psoriasis.

**Figure 1:**
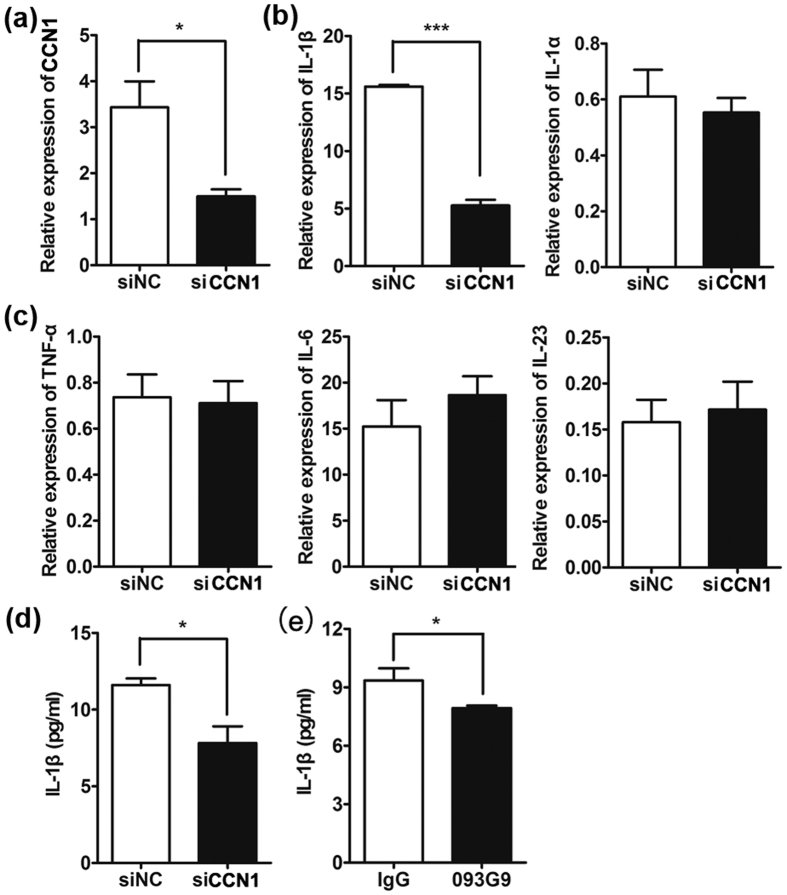
Decreased IL-1β production in HaCaT cells after silencing CCN1 expression. HaCaT cells were treated with specific CCN1 (black bar) or negative control (NC, open bar) small interfering RNA (siRNA) for 24 h. CCN1 mRNA expression (**a**), IL-1β and IL-1α mRNA expression (**b**), TNF-α, IL-6 and IL-23 mRNA expression (**c**) detected by real-time PCR. (**d**) The protein level of IL-1β determined by ELISA. (**e**) CCN1-stimulated production of IL-1β by HaCaT was inhibited by 093G9. HaCaT cells were pretreated with 20 μg/ml of an anti-CCN1 monoclonal antibody (named 093G9, black bar) or control IgG (open bar) for one hour. *P < 0.05, ***P < 0.005. The data represent one of three independent experiments.

To examine the potential role of CCN1 in regulating the production of inflammatory factors, we used a small interfering RNA (siRNA) to knock down CCN1 expression in the KC cell line HaCaT. Using real-time PCR assays, we found a significant reduction in CCN1 expression (Fig. 1a) and decreased IL-1β mRNA levels in CCN1-knockdown KCs, while the level of IL-1α, another member of the IL-1 family, showed no significant change (Fig. 1b). Moreover, the mRNA expression of TNF-α, IL-6, and IL-23 was not altered after knocking down CCN1 (Fig. 1c).

To confirm these results, we examined the protein production of IL-1β in the culture supernatant from KCs by ELISA and found impaired production of IL-1β upon CCN1 knockdown (Fig. 1d). Furthermore, we used a monoclonal antibody, 093G9, to block endogenous CCN1 function in KCs. Similarly, the protein level of IL-1β was substantially decreased in 093G9-treated cells (Fig. 1e). These results suggest that CCN1 altered the production of IL-1β but not TNF-α, IL-6 and IL-23 in KCs, which may contribute to the highly expressed IL-1β observed in lesional skins of psoriasis patients^2^,^11^.

### CCN1 up-regulated IL-1β expression in keratinocytes

**Figure 2:**
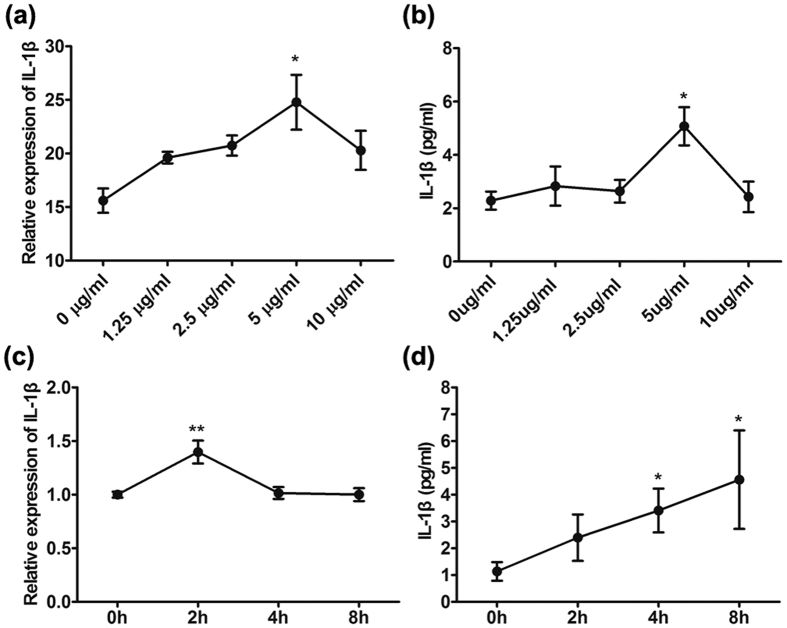
CCN1 increased IL-1β expression in primary cultured adult keratinocytes. The mRNA (**a**) and protein (**b**) levels of IL-1β in keratinocytes stimulated with different doses of CCN1 were detected by real-time PCR and ELISA, respectively. (**c**) IL-1β mRNA expression in keratinocytes treated with CCN1 for 0, 2, 4, and 8 h. (**d**) IL-1β protein production in keratinocytes treated with CCN1 for 0, 2, 4, and 8 h. *P < 0.05, **P < 0.01. The data represent one of three independent experiments.

To further explore whether CCN1 stimulated IL-1β production directly in KCs, we established an *in vitro* cell culture system using primary cultured adult keratinocytes. We first added different doses of recombinant CCN1 protein to the culture and analyzed the mRNA and protein expressions of IL-1β by real-time PCR and ELISA, respectively. The results showed that CCN1 promoted IL-1β production in a dose-dependent manner, and 5 μg/ml of CCN1 had the strongest effect (Fig. 2a,b). Further, we stimulated KCs with 5 μg/ml of CCN1, and the results showed that CCN1 significantly increased IL-1β mRNA expression and reached a peak at 2 h (Fig. 2c). Consistent with these observations, we found elevated IL-1β production in the supernatant of KCs stimulated with CCN1 for 4 h and 8 h (Fig. 2d). We also tested the effect of CCN1 in HaCaT cells, and the results were the same as those in the primary cultured adult keratinocytes (data not shown). These data indicate that CCN1 not only promoted IL-1β mRNA expression but also increased IL-1β protein production in KCs.

### CCN1 increased mature IL-1β release in an endogenous ATP/caspase-1-dependent manner in KCs

**Figure 3:**
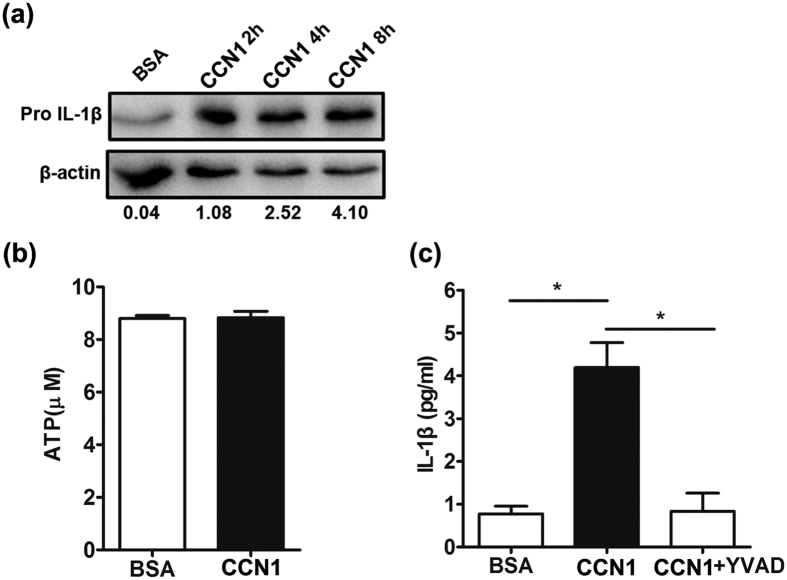
CCN1 promotes mature IL-1β production in an ATP/caspase-1-dependent manner in keratinocytes. (**a**) pro-IL-1β production in keratinocytes stimulated with CCN1 examined by western blotting. (**b**) The level of ATP in keratinocytes determined by an ATP bioluminescence assay kit. (**c**) IL-1β protein production in CCN1-treated keratinocytes after YVAD (1 μM, a caspase 1 inhibitor) treatment. For (**b**,**c**), 5 μg/ml of CCN1 (black bar) or BSA (open bar) was used to stimulate keratinocytes. *P < 0.05. The data represent one of three independent experiments.

Upon stimulation, inactive pro-IL-1β is cleaved into mature IL-1β by caspase-1, and the activation process requires caspase-1 agonists, such as ATP^7^,^16^,^17^. We demonstrated that CCN1 increased IL-1β mRNA expression, and next, we examined the effect of CCN1 on pro-IL-1β production. Using a western blotting assay, we found that CCN1 significantly increased pro-IL-1β production (Fig. 3a). In our previous study, CCN1 could only induce pro-IL-1β production, and exogenous ATP was necessary for the release of mature IL-1β in fibroblast-like synoviocytes (FLS)^29^. However, in this report, CCN1 induced mature IL-1β production directly in KCs (as shown in Fig. 2b). Thus, we examined the endogenous concentration of ATP in KCs using an ATP bioluminescence assay kit, and we detected approximately 9 μM of ATP in the supernatant of KCs (Fig. 3b), which was consistent with the previous report^31^. The results also showed that CCN1 stimulation did not alter ATP concentrations in KCs.

Due to the potent role of caspase-1 in IL-1β post-translational processing, we further explored the function of caspase-1 in CCN1-induced IL-1β expression. We added YVAD (a caspase-1 inhibitor) to CCN1-stimulated KCs and assessed mature IL-1β production. ELISA showed that CCN1-induced IL-1β production was fully inhibited by YVAD in the supernatant of the KCs (Fig. 3c). These results indicate that CCN1 increased mature IL-1β in an endogenous ATP/caspase-1-dependent manner in KCs.

### CCN1 regulated IL-1β production via the α6β1/MAPK p38 signaling pathway

**Figure 4:**
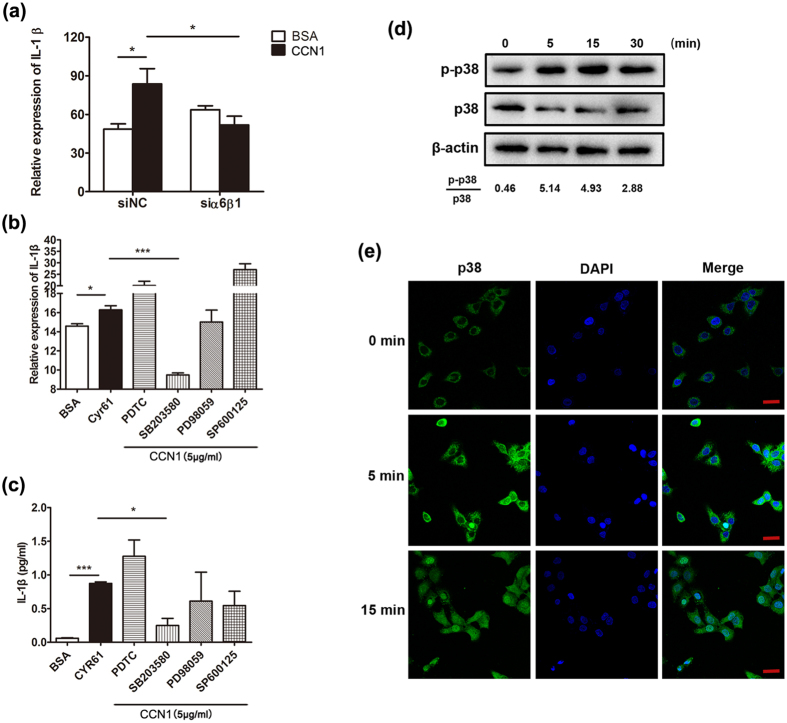
The α6β1/p38 MAPK pathway was activated in keratinocytes. (**a**) IL-1β mRNA in CCN1-administrated HaCaT cells treated with specific α6β1 siRNA. (**b**) The mRNA level of IL-1β in CCN1-challenged keratinocytes. (**c**) The protein level of IL-1β in CCN1-stimulated keratinocytes. For (**b**,**c**), keratinocytes were treated with 4 μM PDTC, 10 μM SB203580, 1 μM PD98059, or 20 μM SP600125 in combination with CCN1 (5 μg/ml) (shadow bars) for 2 h. Open bar, BSA, black bar, CCN1 with no inhibitors. (**d**) Phosphorylation of p38 in CCN1-stimulated HaCaT detected by western blotting analysis. (**e**) Nuclear translocation of p38 in CCN1-stimulated HaCaT cells monitored by immunofluorescence. p38 was detected by FITC-anti-p38 (green). Nuclei were stained with DAPI (4,6-diamidino-2-phenylindole; blue). This merged picture shows p38 translocation to the nucleus. Bar, 20 μm. CCN1 at 5 μg/ml was used to stimulate keratinocytes. *P < 0.05, ***P < 0.005. The data represent one of three independent experiments.

In our previous report, we demonstrated that CCN1 promoted KC activation through the integrin receptor α6β1^27^. To determine whether CCN1 stimulated IL-1β expression by binding to α6β1, we silenced α6β1 production by siRNA. The results showed that IL-1β mRNA expression induced by CCN1 was impaired after blocking the integrin receptor of α6β1 (Fig. 4a).

Next, we used specific inhibitors of ERK (PD98059), JNK (SP600125), p38 (SB203580) and NF-κB (PDTC) to block their kinase activity and examined the alterations in CCN1-induced IL-1β expression. As shown in Fig. 4b, the mRNA level of IL-1β induced by CCN1 was significantly impaired after SB203580 treatment, indicating an important role of p38 MAPK in CCN1-stimulated KCs. IL-1β production in the supernatant of KCs was also decreased by blocking p38 activity in CCN1-treated KCs as determined by ELISA (Fig. 4c). Furthermore, we assessed CCN1-mediated p38 phosphorylation using western blotting analysis. The results showed that CCN1 stimulation rapidly induced p38 phosphorylation, and phosphorylation of p38 peaked at 5–15 min (Fig. 4d).

Then, we examined the nuclear translocation of p38 using scanning confocal microscopy analysis. As shown in Fig. 4e, the transfer of p38 from the cytoplasm to the nucleus increased dramatically after CCN1 stimulation. These data show that CCN1 induced IL-1β expression via the α6β1/p38 MAPK pathway.

### Blockage of CCN1 decreased IL-1β production in psoriasis-like mice

We demonstrated that CCN1 promoted IL-1β expression through the p38 MAPK pathway *in vitro*. Then, we investigated whether CCN1 regulated IL-1β production in mouse models of psoriasis.

**Figure 5:**
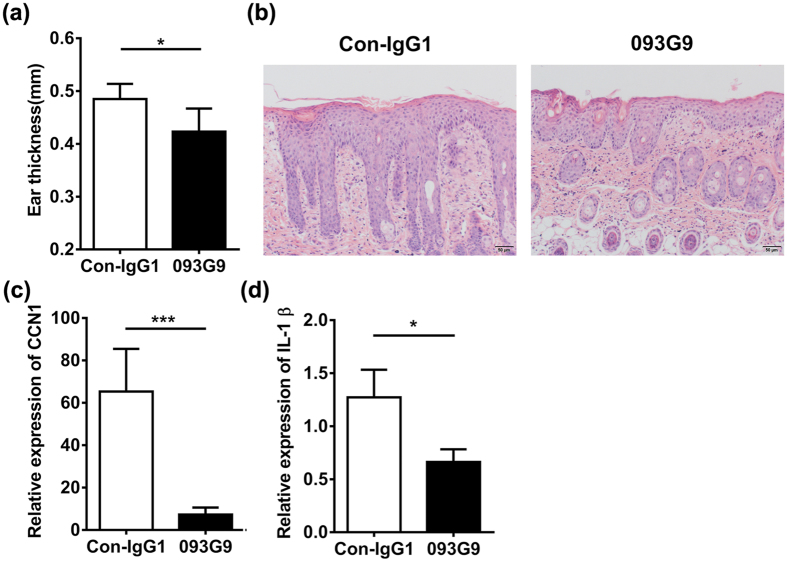
Impaired IL-1β expression in IMQ-induced psoriasis-like mice by blocking CCN1 function. (**a**) Ear thickness of IMQ-induced psoriasis-like mice (n = 6). (**b**) The histopathology of the skin tissue sections of the IMQ-treated mice using H&E staining. Original magnification, 200. Bar, 50 μm. CCN1 mRNA expression (**c**) and IL-1β mRNA expression (**d**) in the back skin of IMQ-induced psoriasis-like mice (n = 6). For (**a**,**c**,**d**), black bar, 1 mg/ml of 093G9; open bar, 1 mg/ml of control antibody (Con-IgG1). *P < 0.05, ***P < 0.001. The data represent one of three independent experiments.

**Figure 6:**
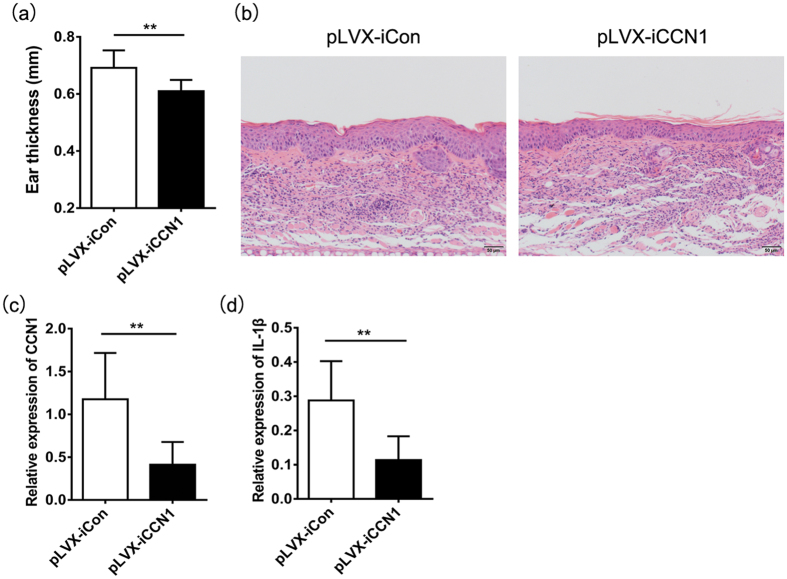
Decreased IL-1β expression in IL-23-induced psoriasis-like mice by knocking down CCN1 expression. (**a**) Ear thickness of IL-23-induced psoriasis-like mice (n = 6). (**b**) The histopathology of the ear tissue sections of the IL-23-treated mice using H&E staining. Original magnification, 200. Bar, 50 μm. CCN1 mRNA expression (**c**) and IL-1β mRNA expression (**d**) in ear skins of IL-23-induced psoriasis-like mice (n = 6). For (**a**,**c**,**d**), black bar, mice treated with pLVX-iCCN1 lentiviral vector. Open bar, mice treated with pLVX-iCon lentiviral vector. **P < 0.01. The data represent one of three independent experiments.

First, we established an IMQ-induced psoriasis-like mouse model and knocked down CCN1 expression with the monoclonal antibody 093G9^27^. After 16 days, the ear thickness of 093G9-treated mice was thinner than that of IgG-treated mice (Fig. 5a). The histopathology data revealed that the epidermis thickness reduced in back skins of 093G9-administrated mice (Fig. 5b). Using a real-time PCR assay, we examined CCN1 and IL-1β expression in the skin of IMQ-treated mice. The results showed that CCN1 and IL-1β expression decreased significantly after 093G9 administration (Fig. 5c,d), which was consistent with the decreased TNF-α and IL-17 expression^27^. Moreover, we used an IL-23-induced psoriasis-like mouse model to confirm these results. CCN1 knock down with a lentiviral vector expressing shRNA (pLVX-iCCN1) significantly decreased the ear thickness (Fig. 6a). The H&E staining data showed decreased epidermis thickness in ears of pLVX-iCCN1 injection mice (Fig. 6b). Furthermore, in ear skin from IL-23-treated mice, the mRNA expression of CCN1 and IL-1β was impaired after blocking CCN1 (Fig. 6c,d), consistent with the impaired TNF-α and IL-17 expression^27^. These data indicate that CCN1 is a mediator of IL-1β production in psoriasis and plays a critical role in regulating inflammation in psoriasis, suggesting that CCN1 could be a potential target for clinical treatment of inflammatory and autoimmune diseases, such as psoriasis.

## Discussion

Psoriasis is a common chronic inflammatory disease that affects the skin and joints^1^,^2^. Although studies have demonstrated that the inflammatory cytokines IL-1β, TNF-α, IL-17 and IL-23 play key roles in the skin lesions and inflammation in psoriasis, extracellular matrix molecules (ECM), such as extradomain A^+^ fibronectin, also promote the skin lesion in psoriasis^32^,^33^,^34^,^35^, indicating that not only IL-1β, TNF-α, IL-17 and IL-23 but also ECM proteins contribute to the pathogenesis and development of psoriasis by up-regulating inflammatory cytokine production. CCN1 is a multipotent factor that contributes to cell proliferation, migration, adhesion, angiogenesis, tissue repair, and tumorigenesis^22^,^23^. Since we first reported the pro-inflammatory role of this protein in RA, CCN1 has been shown to be highly expressed in many autoimmune and inflammatory diseases, such as psoriasis, Sjogren’s syndrome (SS) and systemic lupus erythematosus (SLE)^26^,^36^,^37^. In RA, IL-17 up-regulated CCN1 expression and CCN1 in turn enhanced IL-1β, IL-6 and IL-8 production in FLS, which are involved in the inflammatory niches of autoimmune diseases^24^,^26^,^28^,^29^. However, whether CCN1, a novel pro-inflammatory factor, triggers inflammation in other autoimmune and inflammatory diseases, such as psoriasis, was unknown. We previously showed that over-expression of CCN1 played an important role in promoting keratinocyte hyperplasia in the pathogenesis of psoriasis^27^, and we further explored the contribution of CCN1 to psoriasis inflammation in the current work.

Considering that keratinocytes are the major source of CCN1 in skin^27^, we first used a siRNA to knock down CCN1 expression in HaCaT cells and found that IL-1β expression decreased significantly, while the inflammatory cytokines TNF-α, IL-6 and IL-23 were not altered after CCN1 knock-down (Fig. 1c). In our previous work, silencing CCN1 expression in FLS inhibited not only IL-1β but also TNF-α and IL-6 production^26^,^29^. These results indicate that CCN1 played different roles in epithelial cells and fibroblasts.

Then, we examined the effect of CCN1 on IL-1β production directly. In both primary cultured adult keratinocytes and HaCaT cells, CCN1 increased the mRNA and protein expression of IL-1β (Fig. 2). IL-1β is abundantly present in the lesional skin of psoriasis patients, and a number of cell types in skin are known to produce IL-1β, including monocytes, keratinocytes endothelial cells and fibroblasts^6^,^7^,^38^. As a pleiotropic cytokine, IL-1β can activate various intracellular signaling pathways such as NF-κB, thus promoting downstream effector expression (such as E-selectin, cytokines, chemokines, defensins, ICAM and VCAM)^39^,^40^,^41^,^42^. Most importantly, IL-1 is an initiator of keratinocyte activation^14^,^15^. IL-1 increased the expressions of keratin 6 and keratin 16, which are crucial for initiating and maintaining keratinocyte activation in psoriasis^13^,^14^. Thus, our results indicate that CCN1 not only directly activates keratinocytes but is also involved in the amplification cycle for the chronic inflammation of psoriasis by enhancing IL-1β expression.

Cytokines such as IL-1β, IL-17A, IL-22 and IL-23, which are involved in the pathogenesis of psoriasis, may also contribute to the production of IL-1β. Thus, we examined IL-1β production on primary human keratinocytes stimulated with IL-1β, IL-17A, IL-22 and IL-23. The results showed that only IL-1β and IL-17A could up-regulate the expression of IL-1β, as while IL-22 and IL-23 did not alter IL-1β expression in keratinocytes (Supplementary Figure 1). These results indicated that in keratinocytes, IL-1β production is regulated not only by cytokines such as IL-1β and IL-17A, but also by extracellular matrix protein such as CCN1 in separately independent pathways.

Studies have demonstrated that mature IL-1β production requires two signals: a priming step through pathways such as Toll-like receptor signaling that leads to pro-IL-1β transcription and translation (31 kDa) and a second signal, such as ATP, that results in caspase-1 activation and cleavage of pro-IL-1β into mature IL-1β (17 kDa). Interestingly, we found that CCN1 rapidly increased the mature IL-1β production in the supernatant of KCs, while in FLS, we had to add extracellular ATP to promote the mature IL-1β release. Thus, we examined the concentration of ATP in KCs and found an extremely high level of ATP, which was consistent with other reports^31^. Therefore, in this study, our results showed that CCN1, as a first signal, triggered pro-IL-1β transcription and translation, and endogenous ATP, as a second signal, activated caspase-1 and mature IL-1β release.

Next, we further explored the mechanism of CCN1-induced IL-1β expression in KCs and found that CCN1 regulated IL-1β expression through the p38 MAPK pathway. The MAPK pathway, including p38, ERK and JNK, is critical for cytokine (such as IL-1β, IL-6, IL-8 and TNF-α) production. The signals involved in cytokine expression are different during various conditions. In FLS, we found that CCN1 increased IL-1β expression via AKT/NF-κB but not p38, ERK and JNK, while CCN1 promoted IL-8 expression via AKT, ERK and JNK but not p38^28^,^29^; in keratinocytes, CCN1 stimulation activated p38 only, resulting in increased IL-1β expression, but TNF-α, IL-6 and IL-23 expression was not altered (as shown in Fig. 1b,c). These results indicate that CCN1 increased cytokine transcription using different signals in different cell types. Thus, more studies examining the tight regulation of gene expression (such as post-transcriptional modification) need to be performed.

It has been reported that CCN1 up-regulated IL-1β expression through an unknown mechanism in fibroblasts to promote skin aging^38^, and in DCs, CCN1 could also increase IL-1β production (Supplementary Figure 2). However, as the typical pathogenesis of psoriasis is overactivated keratinocytes proliferation^1^,^2^, the number of dermal DCs and fibroblasts was less than keratinocytes in psoriatic skin lesion. Consistent with our results, KCs are the major sources of CCN1^27^. Thus, CCN1-induced IL-1β expression in KCs might be the first step involved in the pathology of skin diseases *in vivo*. To confirm these results *in vivo*, we further blocked CCN1 with a monoclonal antibody or a lentiviral vector expressing shRNA in IMQ-/IL-23-induced psoriasis-like mice and found impaired IL-1β expression following CCN1 silencing.

In conclusion, the present study reveals a novel mechanism for CCN1 in regulating IL-1β expression via p38 MAPK signaling in keratinocytes, which contributes to the inflammatory microenvironment of psoriasis. This study suggests that the CCN1 protein might be a potential target for reducing inflammation in psoriasis.

## Methods

### Cell culture

Primary cultured normal human keratinocytes were purchased from PromoCell (Heidelberg, Germany). Keratinocytes were cultured using a PromoCell Growth Media Kit in a humidified 5% CO_2_ incubator as previously reported^27^. Keratinocytes were passaged using a Detach Kit (PromoCell) after 80% confluence was reached. HaCaT cells were cultured in a complete high-glucose DMEM medium (HyClone, Logan, UT) containing 10% fetal calf serum (FCS; Gibco, Grand Island, NY), 2 mM L-glutamine, 100 units/ml penicillin, and 100 μg/ml streptomycin in a humidified 5% CO_2_ incubator. In some experiments, 5 μg/ml of CCN1 (Peprotech, Rocky Hill, USA) or 1 μM of YVAD (Santa Cruz Biotechnology, Inc., Dallas, USA), a caspase-1 inhibitor, was added to the cell culture. Additionally, 20 μg/ml of 093G9, a monoclonal antibody that was produced by our group, was used to block CCN1 function in KCs as described in a previous report^26^.

### RNAi knockdown of gene expression

The siRNAs of CCN1 and integrins α6 and β1 were designed and synthesized at Shanghai GenePharma (Shanghai, China), and the sequences are listed in Table S1. The RNAi knockdown of CCN1 expression was described in a previous report^27^. In brief, 2 × 10^5^ HaCaT cells per well were cultured in 24-well plates. After adhesion, a transfection mixture of siRNA oligonucleotides and Lipofectamine reagent (Thermo Fisher Scientific, Massachusetts, USA) in serum-free DMEM medium was added to the HaCaT cells for 4 h. Then, the medium was replaced with complete DMEM containing 2.5% FCS for the subsequent experiments. The mRNA expression of IL-1β, IL-1α, TNF-a, IL-6 and IL-23 was detected by real-time PCR.

### Real-time PCR analysis

Total RNA extraction from KCs and real-time PCR were performed as previously reported^24^. In brief, the mRNA was converted to cDNA using a RevertAid^TM^ First Strand cDNA Synthesis Kit (Thermo Fisher Scientific). A two-step real-time PCR was performed using SYBR Green Master Mix (Applied Biosystems, California, USA), according to the manufacturer’s instructions. Quantitative PCR was performed using a 7500 Fast Real-Time PCR System (Applied Biosystems). The GAPDH (for human)/β-actin (for mouse) gene was used as the endogenous control. The gene expression was calculated as the difference in the cycle threshold (ΔCt) between the target gene and β-actin; ΔΔCt was the difference between the ΔCt values of the test sample and that of the control. The relative expression of the target genes was calculated as 2^−ΔΔCt^. The primers were designed using Primer Express 2.0 software (Applied Biosystems, California, USA) and are listed in Table S2.

### ELISA

The supernatants from KCs were collected, and IL-1β concentration was determined using an ELISA kit (R&D Systems, Minneapolis, USA) according to the manufacturer’s recommendations. A standard curve was performed for each plate and used to calculate the absolute concentrations of the indicated cytokines.

### Western blotting analysis

KCs were lysed, and 30 μg of total protein was loaded for each sample. The expression of pro-IL-1β (31 kDa) was detected with an anti-IL-1β antibody, and the activation of p38 MAPK was analyzed using specific antibodies for total p38 and phosphorylated p38 (Cell Signaling Technology, Beverly, USA) stimulated by CCN1 compared with no CCN1 stimulation. β-actin was used as the endogenous control, and the analysis was performed with Gel-Pro32 software (Gel-Pro Analyzer, Media Cybernetics, Silver Spring, USA).

### ATP bioluminescence assay

The ATP level in keratinocytes was determined with an ATP bioluminescence assay kit (Beyotime, Haimen, China) according to a previous study^29^. In brief, the KC supernatant was collected and added to a 96-well culture plate. Then, 100 μl of an ATP detection working solution was added to each well and incubated for 3 min at room temperature. Finally, the luminescence was measured immediately.

### Analysis of the signaling pathways involved in CCN1-induced IL-1β production

Specific inhibitors of the PI3K/AKT, NF-κB and MAPK signaling pathways (Sigma-Aldrich, St. Louis, USA) were used to analyze IL-1β production following CCN1 stimulation as described in previous reports^27^,^29^. Briefly, 10 μM SB203580 (an inhibitor of p38 MAPK), 1 μM PD98059 (an inhibitor of ERK1/2), 20 μM SP600125 (an inhibitor of JNK) or 4 μM pyrrolidinedithiocarbamate (PDTC; an inhibitor of NF-κB activation) was added to the KCs, and 5 μg/ml CCN1 was also added at same time. Then, expression of IL-1β mRNA was detected by real-time PCR, and the concentration of IL-1β in the KC supernatant was determined by ELISA.

### Immunofluorescence staining for p38 translocation

HaCaT cells were seeded in 24-well plates (2 × 10^5^ cells per well) and stimulated with or without CCN1 (5 μg/ml) for 5 or 15 min. Then, the KCs were fixed with 4% paraformaldehyde and stained with a mouse anti-human p38 antibody (Cell Signaling Technology) and FITC-conjugated anti-mouse antibody (Santa Cruz Biotechnology, Dallas, USA). The samples were monitored with scanning confocal microscopy (Zeiss LSM 800, Jena, Germany).

### Mice

Female BALB/c mice (8–11w old) were purchased from the Shanghai Laboratory Animal Center, Chinese Academy of Science. All experiments were performed according to the Animal Care and Use Committee guidelines of Shanghai Jiao Tong University School of Medicine and the experimental protocols were approved by Department of Laboratory Animal Science, Shanghai Jiao Tong University School Of Medicine. including any relevant details.

For the establishment of IMQ-/IL-23-induced psoriasis-like mice model, the methods had been presented in the section of Supplementary Methods.

### Statistical analysis

All experiments were performed in triplicate. The results are expressed as the mean ± SD. Between-group differences were determined by ANOVA analysis using GraphPad Prism 5.0 (GraphPad Software Inc., San Diego, USA).

## Additional Information

**How to cite this article:** Sun, Y. *et al*. CCN1 promotes IL-1β production in keratinocytes by activating p38 MAPK signaling in psoriasis. *Sci. Rep.* **7**, 43310; doi: 10.1038/srep43310 (2017).

**Publisher's note:** Springer Nature remains neutral with regard to jurisdictional claims in published maps and institutional affiliations.

## Supplementary Material

Supplementary Information
