# Supplementary material for: CCN1 promotes IL-1β production in keratinocytes by activating p38 MAPK signaling in psoriasis
Source: Sci Rep. 2017 Mar 7;7:43310. doi: 10.1038/srep43310 (PMC5339692; doi:10.1038/srep43310)
Supplement: Supplementary Information [file srep43310-s1.doc]

**CCN1 promotes** **IL-1β production in keratinocytes by activating**

**p38 MAPK signaling in psoriasis**

Yue Sun^1, 2^, Jie Zhang^2^, Tianhang Zhai^2^, Huidan Li^2^, Haichuan Li^2^, Rongfen Huo^2^, Baihua Shen^2^, Beiqing Wang^3^, Xiangdong Chen^3^, Ningli Li^2,*^, Jialin Teng^1,*^

^1^ Department of Rheumatology and Immunology, Ruijin Hospital, Shanghai Jiao Tong University School of Medicine, Shanghai, China;

^2^ Shanghai Institute of Immunology and Department of Immunology and Microbiology, Shanghai Jiao Tong University School of Medicine, Shanghai, China

^3^ Department of Dermatology, Ninth People’s Hospital, Shanghai JiaoTong University School of Medicine, Shanghai, China

*Both corresponding authors contributed equally to this work.

Correspondence:

Ningli Li, Shanghai Institute of Immunology and Department of Immunology and Microbiology, Shanghai Jiao Tong University School of Medicine, Shanghai 200025, China, E-mail: ninglixiaoxue57@163.com

Jialin Teng, Department of Rheumatology and Immunology, Ruijin Hospital, Shanghai Jiao Tong University School of Medicine, Shanghai 200025, China, E-mail: tengteng8151@sina.com.

**Supplementary methods**

**RNAilentivirus system**

TheRNAilentivirus system has been described in the previous work [^1^](#_ENREF_1). The pLVX-shRNA2 plasmid was extensively modified to carry a CMV promoter driving the expression of Zs Green1 and the mouse U6 promoter with downstream restriction sites (BamHI and EcoRI) to allow the efficient introduction of oligo nucleotides encoding shRNA (shRNA sequences (1) CCN1 Sense: CCGGCCAGTCCTGCAAATGTAACTACTCGAGTAGTTACATTTGCAGGACTGGTTTTTG; Anti Sense: AATTCAAAAACCAGTCCTGCAAATGTAACTACTCGAGTAGTTACATTTGCAGGACTGG; (2) Control Sense: gatccgTGTTCGCATTATCCGAACCATctcgagATGGTTCGGATAATGCGAACAtttttt; Anti Sense: aattaaaaaaTGTTCGCATTATCCGAACCATctcgagATGGTTCGGATAATGCGAACAcg). The sequence of shRNA targeting CCN1 was designed online (http://www.broadinstitute.org/rnai/public/seq/search).

pLVX-iCCN1 and packaging vectors (pMD2.G, and psPAX2) were co-transfected into HEK293T cells. Titers were determined by infecting HEK293T cells as described [^1^](#_ENREF_1).

**IMQ-induced psoriasis-like mice**

The mice received a daily topical dose of 62.5 mg of a commercially available IMQ cream (5%) (Aldara; 3M Pharmaceutical, St Paul, MN) on ear and the shaved back, translating in a daily dose of 3.125 mg of the active compound. The control mice were treated similarly with Vaseline (Vaseline Lanette cream, Fagron, Rotterdam, the Netherlands).

For the antibody treatment, on day 2 of the IMQ treatment, two groups of mice were applied with anti-CCN1 mAb 093G9 generated in our laboratory or control IgG1 (Millipore, Billerica, MA) at a dose of 200 μg/day i.p., according to previous studies [^2^](#_ENREF_2). On day 16, all mice were sacrificed and skin specimens were collected and inspected.

**IL-23-induced psoriasis-like mice**

The ears of the mice were injected with 20 μl of PBS containing 1 μg recombinant mouse IL-23 (eBioscience Inc., California, USA) or BSA intradermally for 6 consecutive days [^1^](#_ENREF_1). Ear thickness was measured at the center of the ears using a Vernier caliper. For the lentivirus transfection experiment, 2×10^6^ lentivirus particles (20 μl) were injected intradermally into the mouse ears. After 3 days, the mice were treated with IL-23 everyday for 6 days.

For the lentivirus transfection experiment, 2 × 10^6^ lentivirus particles (20 μl) were injected intradermally into the mouse ears. After 3 days, the mice were treated with IL-23 every day for 6 days.

References

1. Sun, Y. *et al.* CCN1, a Pro-Inflammatory Factor, Aggravates Psoriasis Skin Lesions by Promoting Keratinocyte Activation. *The Journal of investigative dermatology* **135**, 2666-2675 (2015).

2. Lin, J. *et al.* Cyr61 induces IL-6 production by fibroblast-like synoviocytes promoting Th17 differentiation in rheumatoid arthritis. *Journal of immunology* **188**, 5776-5784 (2012).

**Table S1** Specific siRNA sequences used in RNAi analysis (human)

| Name | Primer | Sequence (5ˈ-3ˈ) |
| --- | --- | --- |
| Negative Control | FW | UUCUCCGAACGUGUCACGUTT |
|  | RV | ACGUGACACGUUCGGAGAATT |
| CCN1 | FW | CAACGAGGACUGCAGCAAATT |
|  | RV | CAACGAGGACUGCAGCAAATT |
| Integrin α6 | FW | CAGGUUCUCAAGGGUAUAUTT |
|  | RV | AUAUACCCUUGAGAACCUGTT |
| Integrin β1 | FW | GGCUCCAAAGAUAUAAAGATT |
|  | RV | UCUUUAUAUCUUUGGAGCCTT |

**Table S2** Specific primers used in real-time PCR analysis

| Name | Primer | Sequence (5ˈ-3ˈ) |
| --- | --- | --- |
| β-actin (mouse) | FW | TGTCCACCTTCCAGCAGATGT |
|  | RV | AGCTCAGTA ACAGTCCGCCTAG |
| IL-1β (mouse) | FW | CTACAGGCTCCGAGATGAACAAC |
|  | RV | TCCATTGAGGTGGAGAGCTTTC |
| GAPDH (human) | FW | CAACGAGGACUGCAGCAAATT |
|  | RV | TGAGGGTCTCTCTCTTCCTCTTGT |
| CCN1 (human) | FW | GGAACTGGTATCTCCACACGAGTT |
|  | RV | CACCTCACAAATCCGGGTTT |
| IL-1β (human) | FW | TTGTTGAGCCAGGCCTCTCT |
|  | RV | ACCAAATGTGGCCGTGGTT |
| IL-1α (human) | FW | TGGTTCATTTATTCACCCCTTTG |
|  | RV | GCCTTAATCAAGCAAACTTCCCT |
| TNF-α (human) | FW | CCTCTCTCTAATCAGCCCTCTG |
|  | RV | GAGGACCTGGGAGTAGATGAG |
| IL-6 (human) | FW | CCGGGAACGAAAGAGAAGC |
|  | RV | GCGCTTGTGGAGAAGGAGTT |
| IL-23 (human) | FW | CGTCTCCTTCTCCGCTTCAA |
|  | RV | ACCCGGGCGGCTACAG |

Supplementary Figure 1


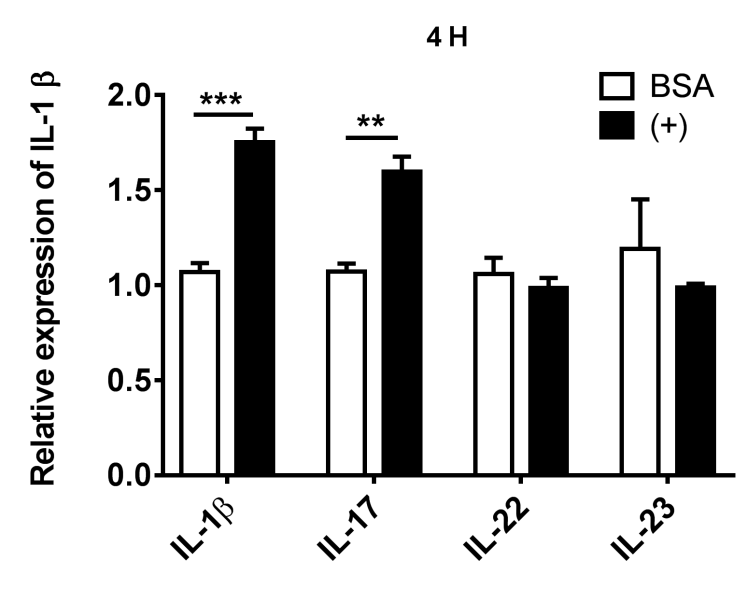


Supplementary Figure 1. The IL-1β expression in primary cultured keratinocytes stimulated by 10ng/ml of IL-1β, IL-17, IL-22 and IL-23 for 2h detected by real-time PCR.

Supplementary Figure 2


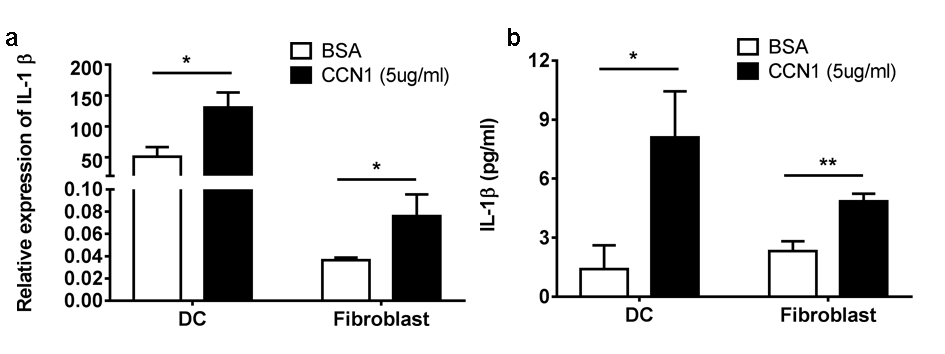


Supplementary Figure 2. The IL-1β expression in primary DCs and fibroblasts stimulated by CCN1 detected by real-time PCR and ELISA.
